# Crowdsourcing contests to facilitate community engagement in HIV cure research: a qualitative evaluation of facilitators and barriers of participation

**DOI:** 10.1186/s12889-020-8185-z

**Published:** 2020-01-15

**Authors:** Yang Zhao, Suzanne Day, Nancy S. Yang, Huanyu Bao, Linghua Li, Allison Mathews, Joseph D. Tucker

**Affiliations:** 1 University of North Carolina at Chapel Hill - Project China, No.2 Lujing Road, Guangzhou, China; 2 School of Social Science, University of Queensland, Brisbane, Australia; 3 Institute for Global Health and Infectious Diseases, University of North Carolina at Chapel Hill, Chapel Hill, USA; 4 University of Minnesota Medical School - Twin Cities, Minneapolis, USA; 5 Infectious Diseases Department, Guangzhou Eighth People’s Hospital, Guangzhou, China; 6 School of Medicine, University of North Carolina at Chapel Hill, Chapel Hill, USA; 7 Faculty of Infectious Diseases, London School of Hygiene and Tropical Medicine, London, UK

**Keywords:** Crowdsourcing contests, Community engagement, Facilitators, Barriers, China

## Abstract

**Background:**

As HIV cure research advances, there is an increasing need for community engagement in health research, especially in low- and middle-income countries with ongoing clinical trials. Crowdsourcing contests provide an innovative bottom-up way to solicit community feedback on clinical trials in order to enhance community engagement. The objective of this study was to identify facilitators and barriers to participating in crowdsourcing contests about HIV cure research in a city with ongoing HIV cure clinical trials.

**Methods:**

We conducted in-depth interviews to evaluate facilitators and barriers to participating in crowdsourcing contests in Guangzhou, China. Contests included the following activities: organizing a call for entries, promoting the call, evaluating entries, celebrating exceptional entries, and sharing entries. We interviewed 31 individuals, including nine HIV cure clinical trial participants, 17 contest participants, and five contest organizers. Our sample included men who have sex with men (20), people living with HIV (14), and people who inject drugs (5). We audio-recorded, transcribed, and thematically analyzed the data using inductive and deductive coding techniques.

**Results:**

Facilitators of crowdsourcing contest participation included responsiveness to lived experiences, strong community interest in HIV research, and community trust in medical professionals and related groups. Contests had more participants if they responded to the lived experiences, challenges, and opportunities of living with HIV in China. Strong community interest in HIV research helped to drive the formulation and execution of HIV cure contests, building support and momentum for these activities. Finally, participant trust in medical professionals and related groups (community-based organizations and contest organizers) further strengthened the ties between community members and researchers. Barriers to participating in crowdsourcing contests included persistent HIV stigma and myths about HIV. Stigma associated with discussing HIV made promotion difficult in certain contexts (e.g., city squares and schools). Myths and misperceptions about HIV science confused participants.

**Conclusions:**

Our data identified facilitators and barriers of participation in HIV cure crowdsourcing contests in China. Our findings could complement existing HIV community engagement strategies and help to design HIV contests for community engagement in other settings, particularly in low- and middle-income countries.

## Background

Curing HIV has become a global strategic priority [1–4], but clinical trials may present significant potential risks to the health of trial participants and raise considerable ethical concerns [1, 5, 6]. Community engagement has been shown to be effective in collecting community input to address ethical issues in health research, including HIV cure research [4, 6, 7]. Community engagement is the process of empowering people to become actively involved in defining and influencing issues that concern them [8, 9]. Community engagement within HIV cure research can enhance communication between researchers, patients, and the community [10, 11], encourage ethical trial participation [1, 6, 12], and assist in post-trial implementation [4].

There has been some HIV cure community engagement in high-income countries (HICs), but less in low- and middle-income countries (LMICs) [10, 13–15]. As HIV cure research advances, there is an increasing need for community engagement, especially in LMIC settings with ongoing clinical trials. One effective way to spur community engagement in public health is through crowdsourcing [13, 16]. Crowdsourcing involves having a large group attempt to solve a problem and then sharing the exceptional solutions with the public [17]. Crowdsourcing challenges explicitly focus on generating public benefit by shifting traditionally individual tasks to large groups [16–18]. One form of crowdsourcing is challenge contests, which involve the following stages: organizing a call for entries, promoting the call, evaluating entries, celebrating exceptional entries, and sharing selected entries [18]. Research and application of crowdsourcing has been limited in both HICs and LMICs [15, 16, 19]. A very limited number of public health projects have used crowdsourcing contests to promote HIV testing [20, 21], encourage condom use [22], and shape health policy [23]. Few crowdsourcing efforts have been conducted in the field of HIV cure research [7, 13].

Two HIV cure clinical trials at the Guangzhou Eighth People’s Hospital, China [24, 25], provided a unique opportunity to both conduct and evaluate crowdsourcing contests. We used crowdsourcing contests to enhance community engagement in HIV cure research by fostering inclusivity and eliciting community perspectives on HIV cure [7, 13]. Crowdsourcing contest provided an opportunity to incorporate the perspectives from a large number of local subpopulations with limited knowledge of HIV cure [7]. However, little research has been conducted to systematically evaluate public health community engagement, including crowdsourcing contests [15, 26]. The objective of this study was to identify facilitators and barriers of crowdsourcing contest participation to promote community engagement in HIV cure research in a city with ongoing HIV cure clinical trials.

## Methods

### HIV cure crowdsourcing contests in Guangzhou, China

The crowdsourcing contests were conducted in Guangzhou city from November 2016 to August 2017, with the goal of understanding the perceptions of HIV cure through crowdsourced contributions. The contests received 471 entries in response to the question, “what would an HIV cure mean to you”, from MSM, PWID, PLHIV and local residents over four months. Contributions were accepted through social media apps and email, as well as in-person at community events, e.g. new year’s party and movie salon. Four hundred and sixty-eight of the entries were texts and three were images. Each participant could choose a small prize less than 1 USD or a raffle entry for an iPad Mini 4. The contest organizers also handed out educational pamphlets to the participants introducing the current state of HIV cure research and basic information about HIV prevention and treatment. From July 2017 to August 2017, the researchers organized three sharing events for MSM, PLHIV, and PWID separately. These events helped to inform contest participants about the research findings of the crowdsourcing contests.

### Community-based participatory research approach

We used a community-based participatory research (CBPR) approach to design the study. We define CBPR as a partnership approach to research that equitably involves community members, organizational representatives, and academic researchers in all aspects of the research process [27]. The CBPR approach is particularly useful when working with marginalized populations because it can facilitate respectful relationships between community members and researchers [28]. We used this approach to engage and solicit inputs from community members at several steps, including writing research concept notes, designing the interview guide, recruiting and interviewing key populations, and coding and analyzing data. These community members received training and functioned in a research capacity. The goal of implementing a CBPR approach in our study was to integrate new knowledge and understanding from the community for the mutual benefit of all partners.

### In-depth interviews

We conducted in-depth interviews to evaluate facilitators and barriers of participation in crowdsourcing contests for HIV cure clinical trial research in Guangzhou, China between October 2018 and December 2018, a year after we implemented the crowdsourcing contests. We used purposive sampling to ensure a range of key populations were engaged, including participants of HIV cure clinical trials and individuals who either participated in the contests or organized the contests. Key populations involved in this study included men who have sex with men (MSM), people who inject drugs (PWID), and people living with HIV (PLHIV). All interviewees were recruited in partnership with a local infectious diseases hospital or two CBOs. Participants were invited to join the study in person or online by a CBO member, a doctor, or research staff. Three pretest interviews were conducted prior to the launch of the study among MSM and PLHIV to develop a standardized interview guide. Our interview guide included the following main questions: demographic information including sexual orientation; perceptions of HIV and HIV cure, crowdsourcing contests, and community engagement; expectations of participating in a contest; participation experience; evaluation of contests; facilitators and barriers to contest participation; and suggestions for future engagement.

All interviews were semi-structured and conducted by a researcher trained in qualitative research techniques at a time and private location of the participant’s choice. All interviews were conducted in Mandarin with a Mandarin-speaking interviewer. The time length of interviews ranged from 32 to 95 min with a median of 60 min. This study was approved by the University of North Carolina at Chapel Hill IRB and the Guangzhou Eighth People’s Hospital IRB. Verbal informed consent was obtained from each individual prior to the interview. We used verbal consent because the study was minor risk and this plan was approved by the IRBs. We audio-recorded and transcribed verbatim all the interviews except one (due to the interviewee’s concerns of privacy), which was recorded using detailed field notes during and immediately after interviewing.

### Data analyses

A CBPR approach was used to analyze the themes of facilitators and barriers. First, all field notes and transcripts were typed and printed to allow for manual coding. Two coders thematically analyzed the data using inductive and deductive coding techniques. We first used an inductive approach to openly code and identify themes that may be related to facilitators and barriers of crowdsourcing contest participation. Then we based our analysis on the results of the first round of coding and drafted a codebook. Two researchers and a CBO worker reviewed the codebook and provided feedback. Two coders then coded the transcripts for a second time based on the revised codebook to deductively identify potential themes. We finalized the codebook based on the results of our second round of coding and conducted a third round of coding to validate our data and conclude the analysis. When coding was complete in each round, discrepancies were addressed and resolved by two coders with the help of four community members, including MSM (2), PWID (1), and PLHIV (1). We evaluated the depth and breadth of community engagement in the crowdsourcing contests by exploring to what extent they had engaged communities based on a CBPR approach [27, 28] and the Good Participatory Practice Guidelines [27].

## Results

**Fig. 1:**
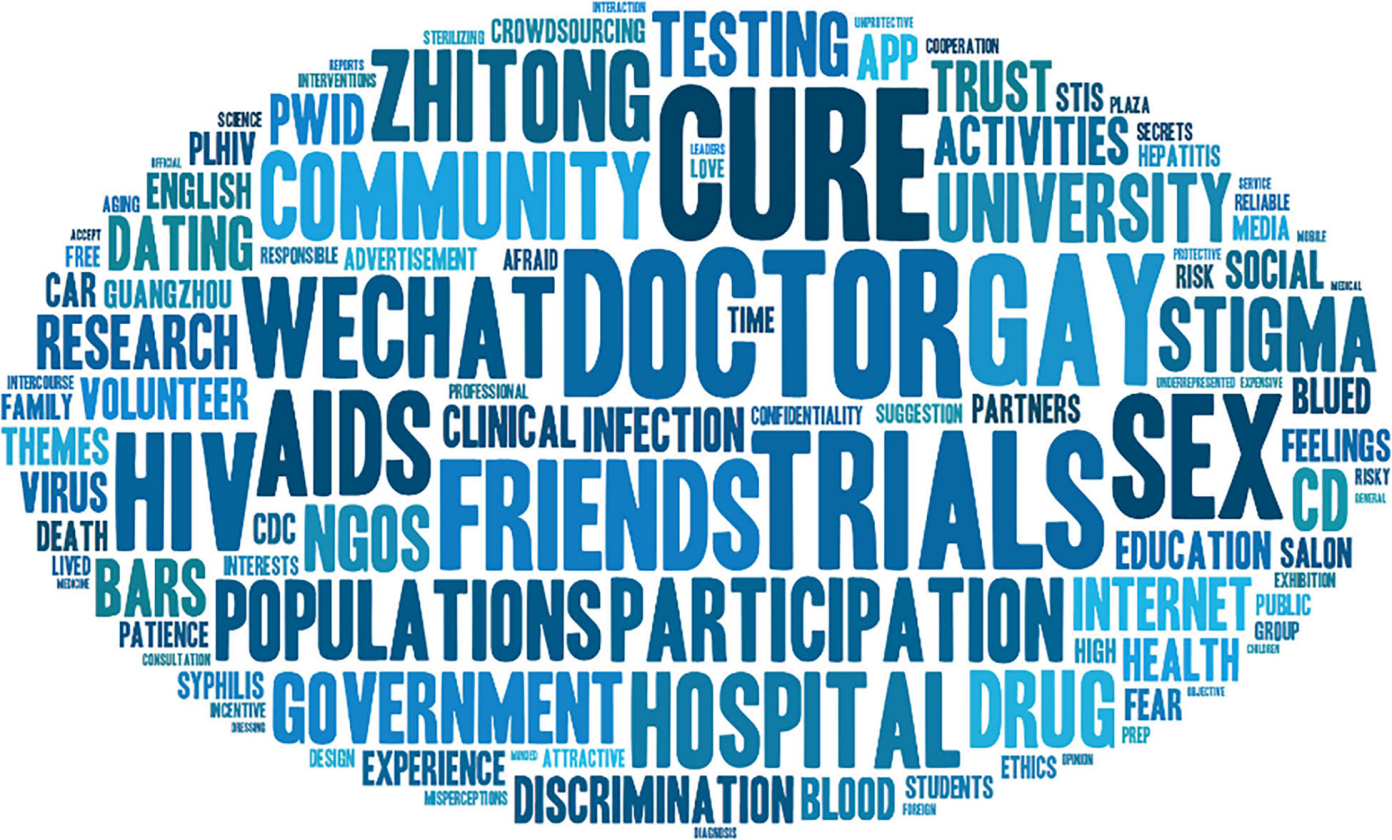
Word cloud of responses from participants. Frequency of usage represented by word size, created by Yang Zhao through www.tagxedo.com

We interviewed 31 individuals (see Table 1 and Fig. 1), including nine trial participants, 16 contest participants, and six contest organizers. Our sample included men who have sex with men (20), people living with HIV (14), and people who inject drugs (5). Facilitators of crowdsourcing contest participation included responsiveness of the contest to lived experiences, high community interest in HIV treatments, and strong trust in medical professionals and related groups (see Table 2). The most frequently mentioned barriers to participation in crowdsourcing contests included persistent HIV stigma and myths and misperceptions about HIV.

**Table 1:** Social-demographic characteristics of interviewees (*N* = 31)

Characteristic		Number of participants (Percentage)
Age	20~29	15 (48.4%)
	30~39	14 (45.2%)
	≥40	2 (6.5%)
Gender	Male	28 (90.3%)
	Female	3 (9.7%)
Sexual orientation	Gay	20 (64.5%)
	Heterosexual	9 (29.0%)
	Nondisclosure	2 (6.5%)
Education level	University and above	20 (64.5%)
	Junior college	4 (12.9%)
	Middle school and below	7 (22.6%)
HIV serostatus	Positive	19 (61.3%)
	Negative	12 (38.7%)
Contest participation	Contest participant	16 (54.8%)
	Contest organizer	6 (16.1%)
	No experience with contest	9 (29.0%)
Trial participation	Trial participant	9 (29.0%)
	No experience with trials	22 (71.0%)

**Table 2:**
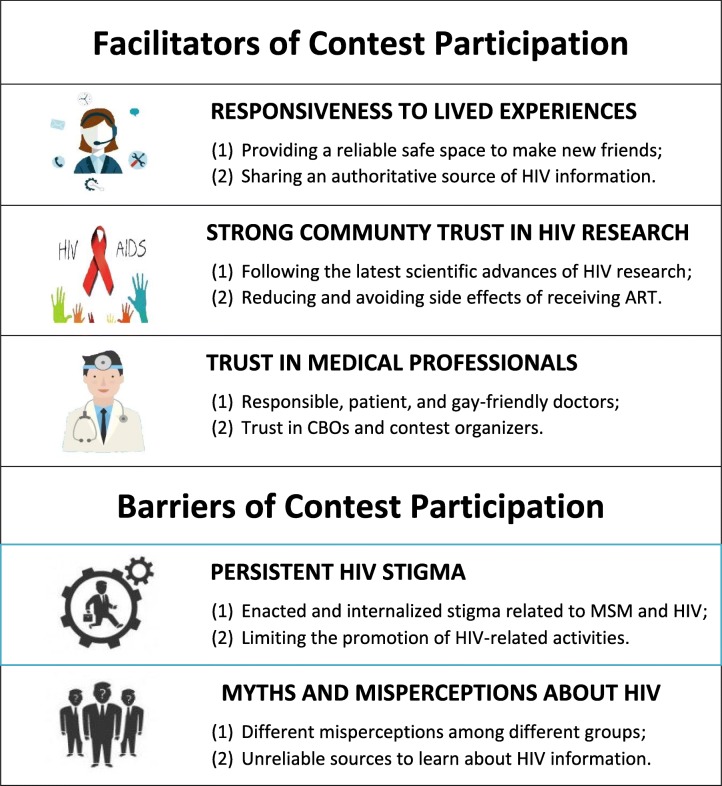
Most frequently reported facilitators of crowdsourcing contest participation.

### Facilitators of participation

#### Responsiveness to lived experiences

Our interviewees reported that they found crowdsourcing contests more compelling when the activities responded to the lived experiences, challenges, and opportunities of living with HIV in China. Crowdsourcing contests were seen as more engaging if they addressed the specific needs of people affected by HIV by empathizing and responding to their lived experiences. For instance, many MSM and PLHIV mentioned their collective experiences of being stigmatized and marginalized in their daily lives. They then expressed a strong desire for a friendly space for MSM and PLHIV to socialize with less fear of discrimination. Our contests were tailored to their needs by providing a reliable safe space where they could socialize and make new friends. We found contests which provided an MSM/PLHIV-friendly space were more successful than those that did not at recruiting targeted populations. As an MSM/PLHIV interviewee said:

“Honestly speaking, taking part in the activity as a gay man, I not only want to listen to the lecture, but also hope to meet and make more friends there, and this is what I really need. It’s very hard to make true friends in my daily life because I have so many secrets.” (No.18, MSM/PLHIV)

Some interviewees also described their experiences of being inundated with misleading HIV news. Most of them felt they were unable to distinguish between news that is valid or invalid, and worried that unreliable sources of HIV information would misshape their perceptions about HIV and affect sexual health-related behaviors. Our contest organizers helped participants identity their own concerns and provided an authoritative and professional source of HIV information, e.g. an educational pamphlet with a stamp of a governmental hospital as a form of expert input to a crowdsourcing contest. As one MSM/PLHIV interviewee mentioned:

“Regarding news of HIV cure clinical trials, I would rather to trust in those from platforms run by hospitals. To me, those platforms were more reliable, professional, and acceptable. Yes, I need a professional source of HIV knowledge to avoid misleading information.” (No.31, MSM/PLHIV)

This preference was also echoed by interviewees who did not identify as being from one of our key populations, expressing how contests helped to address their concerns about safe sexual behaviors and sexual health, as well as the need to provide sexual health education for children.

#### Strong community interest in HIV research

Community interest in HIV research helped build support and momentum for crowdsourcing contest activities and thus facilitated participation. Many interviewees, particularly PLHIV, had a strong desire to keep their knowledge up to date on the latest scientific advances of HIV research through reliable sources. They specifically wanted to learn how to reduce or avoid side effects when receiving antiretroviral therapy (ART) treatment. As one interviewee mentioned:

“As a man who has been diagnosed with HIV recently, I am interested in any information related to HIV, particularly news about HIV cure. If there is a platform introducing the tips to decrease side effects and prolong my life, I would very much like to follow.” (No.16, MSM/PLHIV)

We found that inequitable access to the latest HIV research findings existed among key populations. For instance, HIV research results are usually published in English, which meant that some individuals lacked the necessary skills to find and understand resources in English on the latest HIV research. As one interviewee mentioned, this provided a strong incentive for contest participation:

“Maybe they [MSM] only have a single channel [online group organized by a CBO to learn HIV information]. Unlike me, I never need it. I can always find the information I need via the internet. This may be because those MSM have a relatively lower level of education.” (No.11, MSM/PLHIV)

The interviewees reported that many previous contest participants looked forward to gaining knowledge about HIV from taking part in the contests. Trial participants showed a strong interest in future contests if more professional and recent news on HIV could be provided.

#### Trust in medical professionals

Participant trust in medical professionals and related groups (e.g. CBOs and contest organizers) strengthened the ties between community members and researchers, thus facilitating contest participation. Responses from all HIV cure trial participants and most PLHIV demonstrated their strong trust in medical professionals, including doctors, nurses, and medical students. Responsible, professional, patient, and gay-friendly doctors were most likely to win the trust of key populations. Many participants were introduced to the contests or to contest organizers directly by their doctors. Due to their trust in their doctors, participants accordingly built trusting relationships with the contest organizers, which enabled organizers to promote the engagement activities. As one contest participant mentioned:

“I have built much trust with my doctor through long-term interactions, she always provides me with comprehensive instructions on taking drugs and dealing with its side effects. Trust is so important. If she didn’t invite me, I would possibly not participate in [the contest].” (No.3, MSM/PLHIV)

Some contest participants also described their trust in the CBOs, particularly LGBTQ organizations predominantly serving MSM, social work organizations serving PWID, and the Red Ribbon Society predominantly serving PLHIV. The involvement of CBOs in contest promotion and recruitment increased the willingness of key populations to participate in the contests. As one interviewee reported:

“The social workers working in the methadone clinics have been in contact with the PWID for a long time. I found many PWID trusted them so much. If we collaborated with them in the contests, the PWID would be more willing to join and share their thoughts.” (No.19, contest organizer)

Strong trust in medical professionals, CBOs, and contest organizers constituted an indispensable element for mobilizing community members from key populations to participate in our contests.

### Barriers to contest participation

#### Persistent HIV stigma

Stigma associated with discussing HIV was a barrier to HIV contest participation because it limited the promotion of HIV-related activities in both public settings, e.g. city squares and schools, and private occasions. We identified three types of stigma: enacted stigma related to HIV risk; enacted stigma related to MSM; and internalized stigma related to MSM and/or HIV. Many interviewees reported enacted stigma related to HIV risk as a barrier, which not only prevented them from proactively seeking HIV knowledge, but also reinforced discrimination towards others engaged in HIV-related activities. As one interviewee said:

“Many people become scared once they heard of HIV. They were reluctant to know more about HIV, once they heard of the word ‘HIV’, they would instantly refuse and say, ‘I don’t know.” (No.6, MSM/PLHIV)

Enacted stigma related to MSM also limited contest participation. For instance, we found that some interviewees considered HIV to be a “gay’s disease” that does not affect other populations.

“I felt very strange when I heard of this disease [HIV] initially, why people like us [PWID] have this kind of disease. Weren’t gay men said to be the only population who could be infected with HIV?” (No.9, PWID/PLHIV)

Some individuals internalized the societal stigma directed towards PLHIV. As we found, internalized stigma related to MSM and/or HIV further strengthened these individuals’ reluctance to participate in engagement activities.

“If you are living with HIV, it feels like you have sins. Each time when I came to the hospital, I would wear a mask and a hat. Since I have been positive, I am really unwilling to appear in public.” (No.5, MSM/PLHIV)

We also found mutual stigma and discrimination among members of HIV-affected populations. Some MSM may discriminate against other MSM in a worse economic situation, because they associated lower economic status with higher risk for HIV. Some MSM also believed that interventions should exclude PWID because they viewed PWID as not contributing sufficiently to society.

#### Myths and misperceptions about HIV

Many participants held myths and misperceptions about HIV science that presented a barrier to participation in engagement activities. Among people not living with HIV, they often considered HIV-related issues to be unimportant and unrelated to themselves. Among PLHIV, a common myth was that an HIV cure is as unreachable as ever, and this continue to discourage participation in activities related to HIV cure among key populations and others. As one interviewee mentioned:

“Finding a cure for HIV infection has been a challenge for scientists all over the world. Since I’m neither a scientist nor a medical specialist, I could contribution nothing to the HIV cure research. It makes no sense for me to follow or participate in activities related to HIV cure” (No.25, MSM/PLHIV)

Among PLHIV, we also found that many used unreliable sources to learn about HIV knowledge, e.g. peers they met in the HIV clinic or community-based organizations, social media platforms, and online search engines – all of which may provide misinformation and reproduce HIV myths. As one interviewee mentioned:

“There are many rumors related to HIV and you have to judge their validity. Rumors include an HIV cure or vaccine have been found. You must read many latest materials to distinguish if they are valid.” (No.26, MSM)

Additionally, many individuals had not received formal or comprehensive HIV education and were more likely to be influenced by the existing stereotypes or stigmatization towards HIV. As a result, individuals were deeply vulnerable to myths and misperceptions about HIV and gradually became reluctant to be engaged in any potential HIV-related activities, including crowdsourcing contests.

## Discussion

Based on in-depth interviews among trial participants, contest participants, and contest organizers, we identified several facilitators and barriers to participation in crowdsourcing contests for HIV cure research. Studies have demonstrated that crowdsourcing contests are a feasible strategy for community engagement in HIV cure research [13]. Our findings extend the literature on HIV community engagement and advance our understanding of some factors that may influence participation in HIV cure crowdsourcing contests. Our results may help researchers to design HIV contests for community engagement in other settings, particularly in other LMICs.

We found that trust in medical professionals is an important facilitator of participation in crowdsourcing contests. Previous studies in the United States and China have shown the benefits of patient trust in doctors on enhancing healthcare service, e.g. cultivating patients’ healthy lifestyle behaviors for hypertension [29] and improving ART adherence among HIV patients [30]. However, less research has highlighted the crucial role of medical professionals in community engagement related to public health [31, 32]. We found that doctors not only helped promote contest information and recruit participants directly, but also indirectly attracted large numbers of participants through their visible involvement in the contest engagement activities - for example, when they were involved as a guest speaker. Many participants mentioned that they were introduced or attracted to the contests directly or indirectly by their doctors. These findings are consistent with and extend prior research by showing the specific roles of physicians in promoting participation in crowdsourcing contests as a form of HIV community engagement. Collectively this evidence emphasizes the importance of developing long-term collaborative relations between doctors, patients, and researchers.

Disparities in research literacy and access to accurate HIV information between researchers and the public impacts community engagement efforts. This finding resonates with previous research which has shown low literacy about HIV among key populations as a barrier to community engagement [33, 34]. All barriers frequently reported by our key populations demonstrate that poor HIV research literacy was a major impediment to the latest findings of HIV science. Despite participants’ interest in and desire for up-to-date information on developments in HIV research, limited access to such information reinforced certain misperceptions, e.g. the invalid belief that an HIV cure is as unreachable as ever, further discouraging participation in engagement activities. Our findings suggest that in addition to improved public education about HIV for everyone, improved access to reliable and up to date information about HIV is specifically and urgently needed in order to engage key populations in HIV-related research activities. For instance, HIV researchers could make their findings more accessible to communities by reporting more broadly in non-academic platforms (e.g. blogs, social media) or through open access publications.

We found that stigma constrained participation in crowdsourcing contests. This finding is consistent with prior research which found HIV-related stigma negatively impacts community engagement in HIV research [35–37]. Stigma associated with discussing HIV in public settings was closely connected to broader social inequalities of gender, sexuality, and class. For example, we found male interviewees were more concerned about being incorrectly identified as gay or as having HIV when involved in crowdsourcing contests compared to females. MSM were also worried about disclosing their sexual orientation by participating in an HIV cure contest, and expressed class-based assumptions about HIV risk within the MSM community. The intersectional nature of HIV stigma further complicates community engagement in HIV research. As HIV-related stigma and discrimination is a broad social process of reproducing social differences [38, 39], more efforts are needed to decrease and eliminate social inequalities among community members in order to develop effective strategies to combat HIV stigma.

A main limitation of our study is that we interviewed contest participants a year after we implemented the crowdsourcing contests. This length of time between contest implementation and evaluation may have limited participants’ recollection of their contest participation experiences. To mitigate this concern, we designed a pamphlet including detailed contest information (e.g. the times, locations, and organizers involved in the contests) and also spent time reviewing the contest processes with the interviewees during interviews to help them recall their participation experiences. Second, there may have been some selection bias when recruiting the interviewees. Some participants interviewed for this study were recruited with the help of a doctor who is also a principal investigator of the HIV cure clinical trial. This may have resulted in the inadvertent exclusion of participants who do not have trust in their doctor. However, we also found most contest participants, particularly PLHIV, who were not recruited through their doctor expressed a high trust in doctors as well. Strong trust in medical professionals was still a frequently mentioned facilitator for contest participation across all interviewees. In addition, fewer female were recruited in our study and we were unable to recruit more women, despite additional attempts. Third, we specifically focused on identifying facilitators and barriers to participation in crowdsourcing contests in HIV cure research. The facilitators and barriers to contest implementation are beyond the scope of the current study and require further exploration in the future. However, our findings may be helpful for designing effective strategies of recruitment in future crowdsourcing contests.

## Conclusions

This study identified the most frequently reported facilitators and barriers of participation in HIV cure crowdsourcing contests in China. Our findings could complement existing HIV community engagement strategies and advance our understanding of some factors that may influence participation in HIV cure crowdsourcing contests. Our results may help researchers to design HIV contests for community engagement in other settings, particularly in LMICs.

## Data Availability

The original dataset is owned by the first author. Researchers interested in using these data can contact YZ with specific research questions and a proposal.

## Abbreviations

AIDS: Acquired immune deficiency syndrome
ART: Antiretroviral therapy
CBO: Community-based organizations
CBPR: Community-based participatory research
HIC: High-Income countries
HIV: Human immunodeficiency virus
LGBTQ: Lesbian, gay, bisexual, transgender, and queer
LMIC: Low- and middle-income countries
MSM: Men who have sex with men
PLHIV: People who live with HIV
PWID: People who inject drugs
